# Pattern similarity study of functional sites in protein sequences: lysozymes and cystatins

**DOI:** 10.1186/1471-2091-6-9

**Published:** 2005-05-18

**Authors:** Shuryo Nakai, Eunice CY Li-Chan, Jinglie Dou

**Affiliations:** 1 Food, Nutrition and Health, The University of British Columbia, 6650 Marine Drive, Vancouver, B.C., Canada

## Abstract

**Background:**

Although it is generally agreed that topography is more conserved than sequences, proteins sharing the same fold can have different functions, while there are protein families with low sequence similarity. An alternative method for profile analysis of characteristic conserved positions of the motifs within the 3D structures may be needed for functional annotation of protein sequences. Using the approach of quantitative structure-activity relationships (QSAR), we have proposed a new algorithm for postulating functional mechanisms on the basis of pattern similarity and average of property values of side-chains in segments within sequences. This approach was used to search for functional sites of proteins belonging to the lysozyme and cystatin families.

**Results:**

Hydrophobicity and β-turn propensity of reference segments with 3–7 residues were used for the homology similarity search (HSS) for active sites. Hydrogen bonding was used as the side-chain property for searching the binding sites of lysozymes. The profiles of similarity constants and average values of these parameters as functions of their positions in the sequences could identify both active and substrate binding sites of the lysozyme of *Streptomyces coelicolor*, which has been reported as a new fold enzyme (Cellosyl). The same approach was successfully applied to cystatins, especially for postulating the mechanisms of amyloidosis of human cystatin C as well as human lysozyme.

**Conclusion:**

Pattern similarity and average index values of structure-related properties of side chains in short segments of three residues or longer were, for the first time, successfully applied for predicting functional sites in sequences. This new approach may be applicable to studying functional sites in un-annotated proteins, for which complete 3D structures are not yet available.

## Background

In their recent review of protein sequence analysis *in silico*, Michalovich et al. [1] described the methodology for transferring functional annotation of known proteins to a novel protein. Computer-assisted technology is used to search for and assign the similarity from databases of well-maintained and previously annotated sources. Sequence-based and profile-based searches are conducted using BLAST and PSI-BLAST, respectively. Meanwhile, the Hidden Markov model is more efficient in searching for a distant family. Furthermore, structure-based annotation conducted by using a combination of PSI-BLAST and GenThreader (matching of substitution energy in evolution) may facilitate rapid functional annotation from structure [1]. However, proteins sharing the same fold can have different functions, and structure determination and analysis will not always mean that function can be derived [2]. There are examples of protein families, such as the four-helical cytokine and cytochrome super families, whose sequence similarities are either very low or not detectable [3]. Instead, their topography is more conserved than their sequences. This is rational, since protein functions are classified based on function per se, regardless of whether their sequences or 3D structures are similar or different. An example is the classification of a protein as possessing the function of lysozyme activity, as long as the protein possesses the ability of hydrolyzing peptidoglycans.

Another direct approach for peptide QSAR has been simultaneously investigated in peptide sequence analysis [4]. A critical difference between those two approaches, namely bioinformatics and QSAR, is the prerequisite of 3D structure information on the basis of evolutionary conservation in the case of former; on the other hand, the 3D information is helpful but not always indispensable in the case of latter, by substituting with simpler steric parameters to account for the functional mechanism [5]. For example, Hellberg et al. [4] used altogether 29 properties of side chains of bioactive peptides. After dimension reduction using principal components analysis (PCA) the resultant three main PC scores, i.e., z_1, _z_2 _and z_3, _representing hydrophobicity, molecular size and electronic parameter, respectively, were used as independent variables in regression analysis on the dependent variable of functionality [4].

Meanwhile, by using the homology similarity analysis (HSA), we have found the importance of functional segments within 15-residue sequences of lactoferricin derivatives to correlate with the minimum inhibitory concentration (MIC) [6]. Pattern similarity constant (a correlation coefficient) of the pattern of segments within a test derivative, in comparison to the reference pattern of the corresponding segment and the average of property values of the amino acid side-chains in the most potent derivative, was computed and correlated with MIC of the derivatives. In order to obtain the best (lowest) MIC, the pattern similarity should be close to 1.0 and the average property value should be close to that of the reference potent peptide (template). In the case of the above lactoferricin derivatives, higher correlation coefficients were obtained for log MIC predicted by HSA vs. measured log MIC computed as the output variables of regression ANN (artificial neural networks) than by sequence analysis based on the Hellberg approach [4]. More recently, a different approach, namely "additive QSAR" obtained by substituting with other amino acid residues at different positions in the same sequences, was reported to correlate well with peptide functions [7].

Lejon et al. [8] reported that in PCA analysis of peptide sequences, information of the positions of side chains in the sequence should be included for improving *R*^2^*X *value compared to the results obtained by computing without side chain position data (0.99 vs. 0.60, respectively). Our HSA approach, by segregating segments with and without α-helix propensity, was in good agreement with theirs (*R*^2^*X *of 0.90–0.94 compared to corresponding value of 0.60 but with a much larger number of derivatives). We have further extended this approach to infer the mechanism of emulsifying capacity of peptides with 10–32 residues as a function of hydrophobic periodicity [9]. For the study of emulsification function, a new homology similarity search (HSS) was introduced to plot similarity constants and average property values of segments (3–7 residues) by shifting the segment stepwise from N-terminus towards C-terminus of the sequences; the reference segment used was ELE, i.e., alternate cycle of charged (E), hydrophobic (L) and charged (E) residues. However, emulsification ability is a rather general function of peptides that is not dependent on specific active sites within the sequences; overall, the emulsification ability of peptides was highly correlated with hydrophobic periodicity of their entire sequences.

There are cases of "peptides" which do not have definitive functional sites but requiring specific segments, or "functions" which do require neither specific sites nor segments. The lactoferricin derivatives described in the above study [4] are an example of the former since all of the mutants were prepared as derivatives of the corresponding wild-type lactoferricin 15-residue sequence, which has distinct helical and cationic segments. In contrast, the peptide emulsions [9] are an example of the latter. Typical examples of proteins with definitive functional sites are enzymes, for which the positions of active sites are critical to elucidate the functional mechanisms. Defective protein folding leading to amyloid fibril formation has been associated with various human diseases, such as Alzheimer's and Creutzfelds-Jacob diseases. In 1993, hereditary non-neuropathic systemic amyloidosis was reported to be caused by naturally occurring variants of human lysozyme that aggregated in the liver [10]. Similarly, cystatin C mutation in an elderly man was reported to be the cause of amyloid angiopathy and intracerebral hemorrhage [11].

The recent discovery of a new-fold enzyme named Cellosyl [12] led us to select the lysozyme family in this study as an important one to use for validating the HSS approach to search for functional sites [13]. Meanwhile, loss of papain inhibitory activity in recombinant human cystatin C was reported to be due to insolubilization [14]. Assuming that this loss was induced by an amyloidosis, changes of helix-to-strand in the inhibitory sites as well as the binding sites with papain could also be used for a rational example of application of the HSS approach in this study.

The objective of this paper was to extend application of this new HSS approach to search for functional sites, such as active and substrate binding sites in lysozyme and amyloidosis of cystatin families, to verify the reliability of our new method. The intention was to validate the hypothesis that the evaluation of pattern similarity of short segments with 3–7 residues or even slightly longer in protein sequences is useful in predicting functionality, assuming that they are within allowable topographical units. Accordingly, it is not our intention to replace the 3D approach by the new peptide QSAR proposed in this study; rather, it is anticipated to be supplemental.

## Results

### Lysozyme families

#### PCS classification

**Figure 1:**
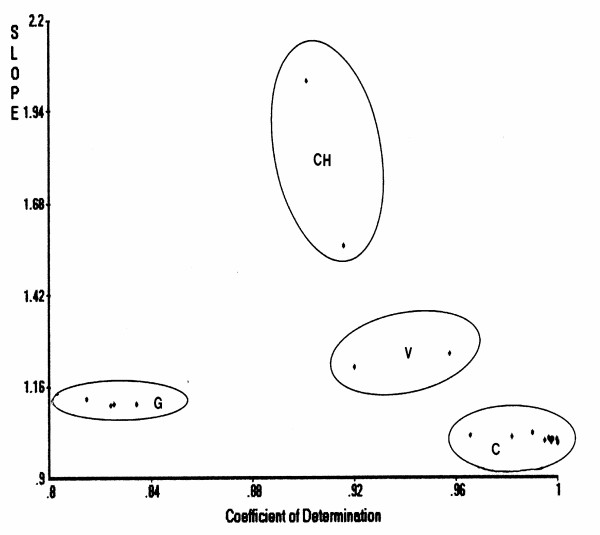
**PCS scattergram of lysozyme families (C-, G-, V-, and CH-types) when hydrophobicity was used as property index. **Hen lysozyme was used as the reference with [coefficient of determination] = 1.0 and [slope] = 1.0.

Figure 1 shows a scattergram derived from principal components similarity (PCS) analysis of 25 lysozyme sequences using the hydrophobicity index of side chains and hen lysozyme as a reference. A scattergram similar to this figure was also obtained when a charge index was used (instead of the hydrophobicity index) for classification. CH-type lysozymes used herein were from a fungus and *Streptomyces globisporus*. The v-type lysozymes were T4 and PA2 phage lysozymes, while g-type lysozymes were from goose, black swan, cassowary, ostrich and chicken G. Sixteen lysozymes (chicken C, human, horse, dog, rat, mouse, red deer, rainbow trout, pigeon, turkey, duck, California quail, Japanese quail, common bobwhite, fruit fly Drosophila, and tobacco hornworm) belong to the c-type family. The large deviations in slope (about 2) of CH-type lysozymes as seen in Figure 1 are suggestive of explicit difference in their molecular structures from those of other lysozyme types.

#### Segment pattern similarity search for active sites

**Figure 2:**
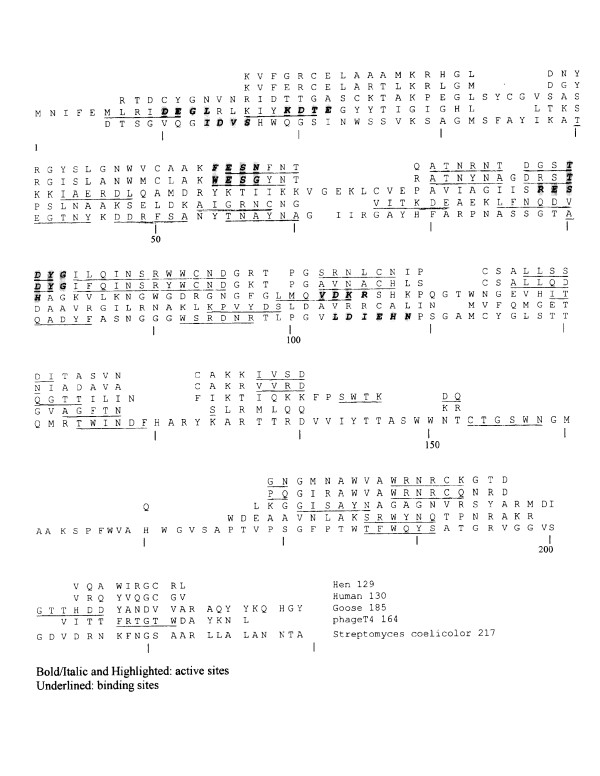
Multiple sequence alignment of five lysozymes belonging to four families.

The HSS computer program was applied to the sequence alignment patterns shown in Figure 2 using the active sites of hen lysozyme at positions 54–57(F^34^ESN) and 80–83(T^51^DYG) as the references. The position numbers in parenthesis are the positions in un-gapped sequences of individual lysozymes, while the immediately preceding numbers outside of parenthesis are the position numbers of the multiple sequence alignment (gapped) in Figure 2. Since the peptidoglycan-lysing activity is associated with glutamic 55(E^35^) and aspartic 81(D^52^) side-chains in the hen lysozyme [13], the segments flanking these residues in the sequences were the focus of pattern similarity search using the HSS.

**Figure 3:**
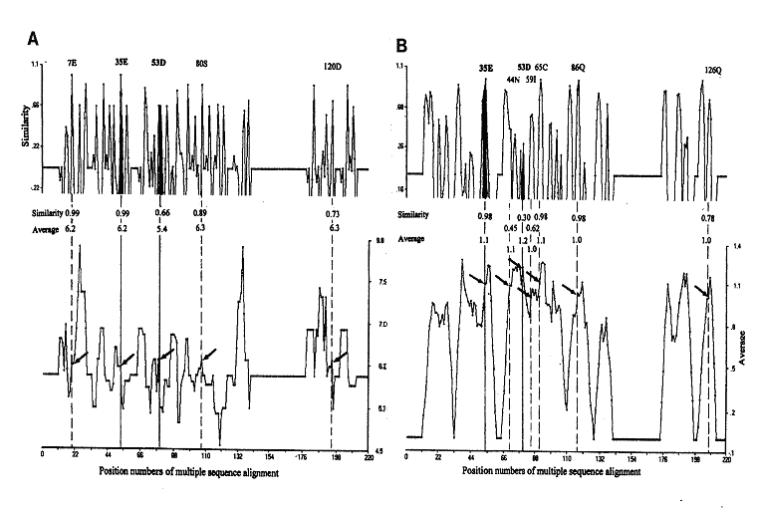
**HSS search patterns for active sites of human lysozyme. **Segment 53–57 of hen lysozyme was used as the reference. A: HSS search pattern based on charge. B: HSS search pattern based on turn propensity.

The search patterns illustrated in Figure 3A (charge) and 3B (turn propensity) are a trial run to validate the HSS approach, applied to human lysozyme vs. hen lysozyme, employing the five residues flanking E^35 ^(K^33^FESN of hen) as a search unit. The rules herein for selecting active segments are that the greater the similarity approaching to 1.0 and the nearer the average value to that of the reference, the more likely to be active sites in the test sequence. In Figure 3A, in addition to E^35 ^and D^53^, three residues of E^7^, S^80 ^and D^120 ^show about the same similarity constants (upper pattern) as well as similar average charge values (shown by arrows on the lower pattern). However, none of the above latter three positions are acceptable as the active site, as N^44^, I^59^, C^65^, Q^86^, and Q^126 ^in Figure 3B for β-turn search do not have matching peaks in Figure 2A for charge search. Therefore, only E^35 ^and D^53 ^are qualified as the active positions of human lysozyme. The same result was obtained when five residues flanking D^52 ^(S^50^TDYG) of hen lysozyme were used as an alternative reference segment. The two regions around E^35 ^and D^53 ^determined for human lysozyme are in good agreement with those being reported for the active site in the literature [13]. Despite the fact that the charge is of prime importance in defining active sites of lysozymes, the turn propensity values of the segment rather than its pattern similarity appear to play an important role in the enzyme, as exemplified in a low similarity value of 0.30 for D^53 ^compared to 0.98 for E^35^, whereas similar average turn values of 1.2 and 1.1, respectively, were computed (Fig. 3B). All these results may imply that the exposure of active sites to react with the substrate binding sites is essential.

**Figure 4:**
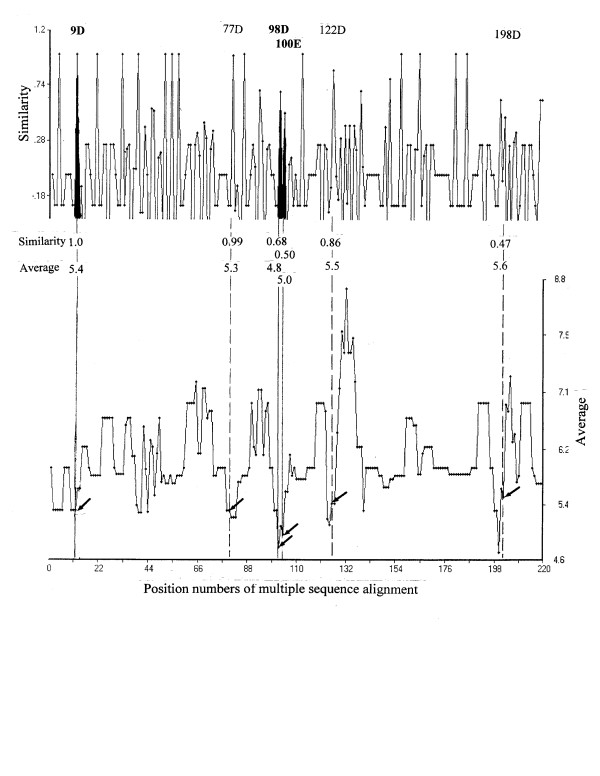
**HSS search patterns for active sites in *Strep. coelicolor *lysozyme. **Segment 79–83 (STDYG) of hen lysozyme was used as the reference based on charge.

The same search was conducted for goose and T4 lysozymes as well as the new fold CH-lysozyme [12], with the assumption that it is an un-annotated sequence, to confirm validation of the HSS approach (Table 1). Two positions of 79(E^73^) and 103(D^97^) were determined as the active sites of the goose lysozyme sequence. D^97 ^may not be essential for the catalytic activity of the goose lysozyme [15]. In comparison, 11(E^11^) and 20(D^20^) were noted for T4 lysozyme, which are in good agreement with those reported in the literature [13]. As shown in Figure 4, in the case of the sequence of Cellosyl (CH-type), 14(D^9^), 104(D^98^) and 106(E^100^) were identified as candidates to be the active positions of catalysis, which are in good agreement with Rau et al. [12]. These results would support the reliability of the HSS approach.

**Table 1:** Determination of active sites in sequences of lysozymes in different families

Family	Active sites	Position	Charge 1	Charge 2	Turn 1	Turn 2
Hen	1	55(35)E	1.0/5.0	.98/5.0	1.0/1.12	
	2	81(52)D	.98/5.2	1.0/5.2		1.0/1.21
Hman	1	55(35)E	1.0/5.9	.98/5.0	.98/1.17	
	2	81(53)D	.98/5.2	1.0/5.2		1.01/1.22
Goose	1	79(73)E	.67/6.9	.78/6.9	.06/1.03	
	2	103(97)D	.83/7.3	.83/7.3		.67/,92
T4	1	11(11)E	.61/4.5	.55/4.5	.76/1.16	
	2	20(20)D	.55/5.4	.55/5.4		.53/1.07
CH	1	14(9)D	.98/5.2	1.0/5.2		.95/.95
	2	104(98)D	.74/4.5	.65/4.5		.81/.92
	3	106(100)E	.95/5.6	.86/5.6		.87/.97

Position: the first number is the multiple sequence alignment position shown in Figure 2. Number in bracket is that in the sequence of each lysozyme. Charge 1 and 2 and Turn 1 and 2: Sites 54–57 and 80–83 (alignment position numbers in Fig. 2) were used as references. These position numbers correspond to 34–37 and 50–54, respectively in the sequence of hen lysozyme. The first and second digits n each cell of Charge and Turn are similarity constant and average of property value.

#### HSS search for substrate binding sites

Compared to the active sites that are explicitly negative in charge at single positions, the substrate binding sites of lysozymes are rather loosely defined, mainly due to the greater complexity of determining the 3D structure of enzyme-substrate complexes than that of enzyme alone. It is generally agreed that the substrates locate inside the cleft formed between the helix lobe and the strand lobe of most lysozyme molecules, except for Cellosyl, the bacterial muramidase from *Streptomyces coelicolor*, which can be attributed to structural difference in the catalytic crevice [12]. The six-residue segment at alignment positions 84–89 (I^55^LQINS) of hen lysozyme was employed as the reference segment [16] using the hydrogen bonding scale (Table 2). Based on the high pattern similarity constants and the average hydrogen bonding index values, eight potential sites were identified for hen and human lysozymes. The same rule as that for selecting active site was used herein for selecting of binding sites. Position 77(D^48^) and 188(W^111^) shown in "Hen 1" of Table 2 with lower similarity constant and hydrogen bonding value, respectively, may not be the potent substrate binding sites in the hen lysozyme. In the 3D structure [17], those two positions are far away from the catalytic cleft where the substrates snugly fit in. In the human lysozyme, 188(W^112^) is more likely to be the binding site than 102(A^73^), with higher pattern similarity and strength of hydrogen bonding of 0.89/0.45 than 0.80/0.32, respectively. Not only strong hydrogen bonding, but also high pattern similarity of a segment may be required to be qualified for substrate binding sites.

**Table 2:** Determination of substrate binding sites in amino acid sequences of lysozyme families

	**Potential sites**								
Hen 1	**55(35)E**	**70(42)A**	77(48)D	**84(55)I**	**90(61)R**	**102(72)S**	**117(83)L**		118(111)W
	**.81/.71**	**.79/.53**	.34/.65	**1.0/.41**	**.56/.58**	**.78/.56**	**.61/.42**		.66/.35
									
Hen 2	**36S**	**45R**	**49G**	**56L**	**62W**	**73R**	**90A**	**100S**	112R
	**.82/.71**	**.86/.44**	**.79/.47**	**1.0/.39**	**.59/.58**	**.91/.47**	**.98/.41**	**.55/.46**	.76/.29
									
Human 1	**54(34)W**	**70(42)A**	77(49)D	**84(56)I**	**90(62)R**	102(73)A	**116(83)A**		**118(112)W**
	**.48/.52**	**.42/.51**	.34/.65	**.91/.47**	**.50/.61**	.80/.32	**.61/.46**		**.89/.45**
									
Human 2	27N	**43T**	**50R**	**57F**	**63Y**	74V	84L	101R	**117Q**
	.56/.39	**.56/.51**	**.61/.47**	**1.0/.45**	**.55/.61**	.72/.33	.48/.47	.58/.29	**.79/.45**
									
Goose	43(73)I	99(93)L	**119(113)I**	**143(133)S**	181(143)G	**201(163)G**			
	.85/.31	.76/.34	**.67/.46**	**.81/.55**	.82/.36	**.81/.58**			
									
T4	**6(6)M**	**16(16)K**	**53(49)A**	**66(57)V**	**75(66)L**	94(85)K	**123(112)A**	**186(136)S**	207(153)F
	**.74/.34**	**.99/.38**	.78/.30	**1.0/.42**	**.52/.54**	.67/.38	**.73/.45**	**.58/.51**	.78/.36
									
CH	**40(35)T**	**47(42)D**	**55(50)T**	80(74)A	**92(86)W**	**124(118)T**	153(147)C	186(180)T	
	**1.0/.58**	**.63/.52**	**.80/.51**	.51/.44	**.73/.49**	**.83/.59**	.51/.56	.56/.54	

The first/second digits are similarity constants and average hydrogen bond index values of binding site with segments of sox residues beginning with the positions shown. Bold digits show more likely binding sites than non-bold digits.

Hen 1 and Human 1: from the alignment shown in Figure 2; Hen 2 and Human 2; from the alignment of the lysozyme C family exclusively.

For the goose lysozyme, six potential binding positions were detected (Table 2); I^113 ^and G^163^appear more likely to be the binding sites than other positions considering the location of the cleft in the molecule. Similarly, nine sites were found to be potential sites of T4 lysozyme, especially three positions, i.e. M^6^, L^66 ^and S^136 ^(Table 2). In the case of Cellosyl, eight positions were identified as the potential binding sites (Table 2). Probably due to the considerable 3D-structure difference of this lysozyme from those of other lysozyme families [12], the alignment positions 40–45 (T^34^EGTNY) instead of hen's 84–89 (I^55^LQINS) were used as a reference segment for obtaining more rational search results. Instead of hydrogen-bonding motivated interactions, less polar van der Waals interaction with the aromatic side chains in CH-lysozyme may be regarded as the second important stereochemical forces in the substrate binding [16].

In the literature, the most frequently cited substrate-binding sites in c-type lysozymes family have been W^62 ^and D^101 ^of hen lysozyme [13]. Since Figure 2 includes the distant family of CH-type, the segment similarity search was repeated within the c-type lysozymes alone to restrict the search within similar fold. The results are shown as "hen 2" and "human 2" in Table 2. Those results almost perfectly match to the substrate binding mechanism based on X-ray crystallographic analysis, e.g. D^101^, N^103^, N^104^, A^107^, V^109^, E^35^, N^46^, V^110^, E^52^, N^59^, and W^63 ^[15]. Almost all of these side-chains are very close or adjacent to the segments listed in "Hen 1" and "Hen 2" of Table 2.

#### Substrate binding sites reported by site-directed mutagenesis

Among three mutants obtained by replacing W^62 ^with Y, F or H, the W62H mutant, and especially the double mutant W62H/D101G, reduced substrate binding drastically [15]. This change can be explained by a decrease in the hydrogen bond average value from 0.58 to 0.54 and from 0.46 to 0.29 in V62H and D101G, respectively, when the index values employed in this study were used in computation. The double mutant changed substrate-binding mode while maintaining the overall protein structure almost identical to that of the wild type [18]. An extensive cluster of hydrophobic structure is involved in distinct regions of the sequence, but is all disrupted by a single point mutation of W62G located at the interface of the two major structural domains in the native lysozyme [19]. Similar effects were observed in mutants Y63L and D102E of human lysozyme [20]. The double mutants R41N/R101S and V74R/Q126R of human lysozyme were better catalysts for lysis of *Micrococcus lysodeikticus *[18]. The average hydrogen bond value of both R41N and R102S was shown to increase in our HSS search, but similar effects could not be observed for V74R/Q126R. An interesting finding is that these two mutations have both resulted in the side chains being identical to those of hen lysozyme. R^41 ^and V^74 ^are near A^42 ^and A^73^, respectively. Importance of R^115 ^in substrate binding of human lysozyme was reported [21], which is in good agreement of W^112 ^within the same subsite F (Table 2).

### Cystatins

#### PCS classification

**Figure 5:**
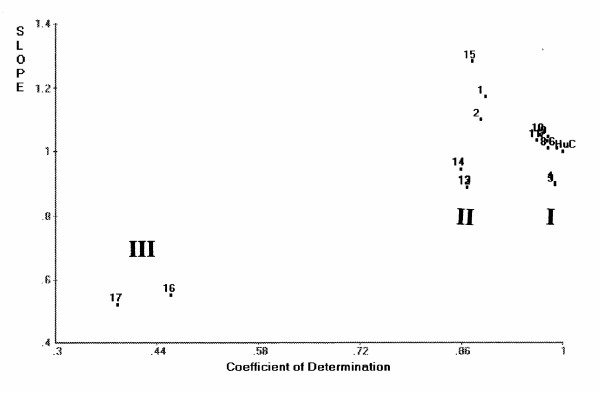
**PCS scattergram of cystatins when hydrophobicity was used as property index. **Human cystatin C (HuC) was used as the reference. Human cystatins M, E, S, SA, SN, D, F, A and B are labelled as 1, 2, 7, 8, 9, 10, 15, 16 and 17. Labels 3, 4, 6, and 11–14 are for cystatins from mouse C, rat C, bovine, hen, rainbow trout, chum salmon and carp, respectively.

The PCS scattergram of the 17 cystatins using human cystatin C (HCC) as a reference and hydrophobicity as side-chain property index is shown in Figure 5; alteration of the index to α-helix and β-strand propensities did not appreciably change the grouping results. The three groups include human cystatins C, D, S, SA, SN and hen cystatin (Group I), human cystatins E, F and M (Group II), and human cystatins A and B (Group III) which are the stefin group cystatins that are smaller in molecular size and have slightly lower papain inhibitory activity than HCC [22].

#### Active sites

**Figure 6:**
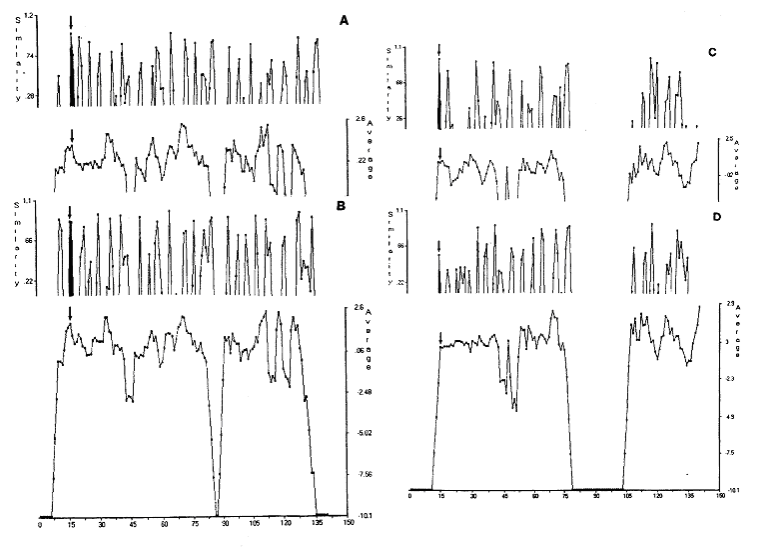
**HSS search patterns for active sites of cystatins against human cystatin C based on hydrophobicity. **A: HCC (reference), B: EWC (egg white cystatin), C: HCA and D: HCB.

The HSS patterns using hydrophobicity to search for active sites of the four cystatins (HCC, EWC, HCA and HCB), when L^9^VGG of HCC has been employed as the reference, are shown in Figure 6. The active sites shown with arrows are at about the same location in all cystatins sequences used in this study. Similar results were obtained when bulkiness was used as side-chain index. In these cases, the similarity peaks are the major clue for identifying active sites. However, when similarity peaks appear in the neighbourhood, the average index values would become more reliable for identifying active sites as shown in Figure 6B; hen cystatin has two probable active positions side by side with the same similarity constants. The active sites, therefore, should be near the N-terminus with Prosite-type patterns of [L,I,M]-x(4)-G- [G,A]; the active sites are L^9^VGG and L^7^LGA in human cystatin C and hen cystatin, respectively. The similarity constants and the average hydrophobicity of the active sites are shown in Table 3. Stefin B (HCB) shows much lower similarity constant and average hydrophobicity than those of other cystatins. This result is in good agreement with *k*_*i *_difference reported by Abrahamson [23].

**Table 3:** Active and substrate binding sites of cystatins

	**HCC**	**EWC**	**HCA**	**HCB**
**Active site**				
Segment	L^9^VGG	L^7^LGA	I^2^PGG	M^2^SGA
SimConst/Av.Hydroph*	1.00/1.35	0.87/1.80	0.97/1.00	0.55/-0.01
**Binding site 1**				
Segment	Q^55^IVAG	Q^53^LVSG	Q^46^VVAG	Q^46^VVAG
SimConst/Av.Hydroph	1.00/0.90	0.70/0.92	0.90/0.95	0.90/0.95
**Binding site 2**				
Segment	V^104^PWQG	I^102^PWLN	L^73^PGQN	L^73^PHEN
SimConst/Av.Hydroph	1.00/1.01	0.35/1.95	0.30/0.98	-0.16/0.42
SimConst/Av.Turn**	1.00/1.05	0.90/0.96	0.56/1.22	0.99/1.06

* Pattern similarity constant / average hydrophobicity

** Pattern similarity constant / average turn propensity

Similar to the corresponding tables for lysozymes (Tables 1 and 2), Sim/Const is 1.0 for reference HCC, and the greater the Av.Property the greater the property strength.

#### Substrate binding sites

A HSA study similar to our previous paper [6] was conducted at the active and two binding sites of cystatins, yielding results (Table 3) which are in good agreement with Turk et al. [22]. Substrate-binding site 1 had the pattern Q-x(3)-V- [S,A]-G, while substrate-binding site 2 had the pattern [L,I,V]-P-x(3)-x(3)- [N,G]. Similarity constants of binding loop 2 of egg white cystatin (EWC) and HCA are lower than that of HCC, whereas not only similarity but also average hydrophobicity are lower in HCB. Similarity constants at the active site (against 1.0 for HCC) using hydrophobicity index were >0.8 for cystatins A, D, F and hen, ~0.5 for E and M, and 0.1–0.2 for cystatins B, S, SA and AN. Similarity constants at binding loop 2, when strand propensity was used for PCS computation, were lower for stefins A and B with values of 0.8 and 0.6, respectively, than >0.9 for other cystatins. On the other hand, similarity constants for strand at binding site 1 were not much different among different cystatins, with values >0.9 (not included in Table 3).

It is interesting to note that stefins A and B do not have the PW pair which is in the binding site 2 of HCC and EWC; instead they have PG and PH pairs, respectively (Table 3). The W → G replacement increased strand propensity, while W → H replacement did so moderately. The values shown in Table 3 were almost inversely proportional to the equilibrium inhibition constant *k*_*i *_except for human cystatin S that was weak in the inhibitory activity, which might have been due to the difference in phosphorylation of serine at N-terminal region [23]. Although stefins A and B are classified differently from other groups on PCS scattergram (Fig. 5), the weak binding at the binding site 2 may not have considerable effects on the *k*_*i *_values.

Turk et al. [22] have stated that the differences in the binding constants between cystatins and various cysteine proteases arise primarily from differences in the structure of enzyme active site clefts. The inhibition of endopeptidases, i.e. papain and cathepsins S and L, by cystatins is extremely tight and rapid, whereas the inhibition of exopeptidases, i.e. cathepsins B and H, is considerably weaker. The active site cleft of known endopeptidases is free to accommodate inhibitors, while in the case of exopeptidases, the active site cleft contains extra residues in it. In the N-terminal region of cystatins, it was observed that the affinity for target proteases decreased with both size and charge of substituting residues [22]. These observations are in good agreement with the results when bulkiness of side chains was used for the HSS computation for the binding site 1, "SimConst /Av.bulk" values were 1.00/12.3, 0.92/14.2, 0.98/11.4 and 0.91/11.34 for HCC, EWC, HCA and HCB, respectively. As expected, stefins A and B were less bulky. Furthermore, HCC and EWC include longer chains at the N-terminal sides with bulkier residues than those of stefins. These findings are in good agreement with the effect of bulkiness of G^4 ^in the stefin A sequence, implying that the bulkier the residue at position 4, the weaker the papain inhibitory activity [24].

### Amyloidosis

#### Lysozymes

Similarity constants and average propensities of α-helix and β-strand were computed for G^54^IL and G^54^ILQIN of hen lysozyme (G^55^IF and G^55^IFQIN in the case of human lysozyme) as shown in Table 4. The amyloidogenic mutant I55T showed increased strand propensity from 0.82 to 0.85, without a substantial change in the similarity constants. With regard to the helix structure, the similarity constant decreased from 1.00 to 0.93 while the average value increased from 1.08 to 1.14, thereby implicating a decrease in helix, since the helix index used herein is inversely related to the content of helix structure. These changes are favourable for amyloidosis. The fact that position 56 in human lysozyme is near its active position at D^53 ^may explain its dramatic effect on the enzymatic activity, more effective than other positions in the sequence. The six-residue computation did not show as clear a difference as was found in the three-residue computation. Nine double-site mutations in addition to I55T at other positions selected at random in the sequences of the amyloidogenic mutant I55T by using the RCG program did not restore the activity of wild-type lysozyme (unpublished). These results infer that the amyloid, once formed by detrimental mutation at the active site, cannot be restored by mutation at other locations in the sequence.

**Table 4:** HSA computation for I55T lysozyme

	**Hen**	**Human**	**I55T **(Hen)
**Helix**			
G^54^IL(F)QINS			
SimConst*	1.000	0.917	0.952
Average	0.960	0.993	0.985
G^54^IL(F)			
SimConst	1.000	1.000	0.927
Average	1.082	1.082	1.141
**Strand**			
G^54^IL(F)QINS			
SimConst	1.000	0.978	0.997
Average	0.788	0.762	0.797
G^54^IL(F)			
SimConst	1.000	1.000	0.998
Average	0.822	0.822	0.845

L^56 ^for hen and F^57 ^for human. * Similarity constant.

#### Cystatins

**Figure 7:**
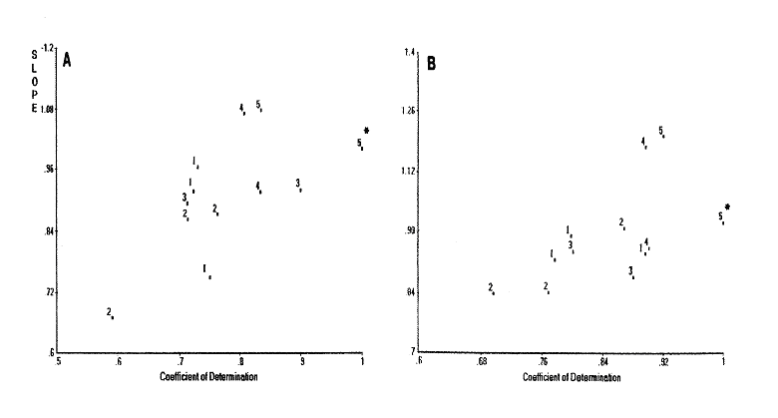
**Effects of mutating the strand domain of human cystatin C (mutated 21 residues). **A: Strand index, B: Helix index. The numbers show multiples of activity increases from wild-type. 1 shows no increase of inhibitory activity. 5* is the reference mutant 12W86V with the highest inhibitory activity.

Heat treatment of HCC induced its dimer formation at an early stage of separation, resulting in a complete loss of its activity [25]. Based on a dramatic decrease in the monomer form as shown by its CD spectrum, polymerization such as amyloidosis could be a cause of the loss of papain inhibitory activity of mutated HCC [14]. Of 35 residues (positions 1–35) of the helix domain of HCC, 17 residues were mutated in the 22 single-site mutants using the RCG program [14 (Table 1)]. When 33 mutants obtained by adding one extra residue each in both side of the original 17 residues after eliminating duplication were used for PCS computation, the resultant PCS demonstrated that helix propensity and bulkiness were playing important roles in thermostability (data not shown). Employment of three residues flanking the mutated residue was important in pattern similarity computation. This conclusion is in good agreement with Hall et al. [26] who did an exhaustive study showing that mutations at positions 8–10 enhanced thermostability of cystatin. With regard to the papain inhibitory activity, the importance of hydrophobicity and bulkiness was demonstrated (the PCS scattergrams, similar to Fig. 7, are not shown here).

Of 86 residues in the strand domain (positions 36–121) of HCC, 21 residues (positions 36–120) were mutated in the 23 double-site mutants using the RCG program [14]. Thirty-seven residues were used for the PCS computation of single-site mutations as described above. Hydrophobicity appeared to be playing an important role in thermostability, while strand propensity was important for inhibitory activity (data not shown). Strand and helix propensities in the strand domain were influential to the papain inhibitory activity of HCC (Fig. 7). The figures show 12 data points only by eliminating data from single site mutation in the helix domain, which did not show distinct trends with broader scatter in these figures. The second mutations in addition to the above single mutations were conducted at the strand domain of the enzyme [14]. Coefficient of determination of 1.0 and slope of 1 indicate perfect match with the reference sample (5*) that is mutant G12W/H86V with the lowest strand propensity along with highest helix propensity in the strand domain among 23 double mutants. It is worth noting that the PCS is a classification program comparing pattern similarity without demonstrating quantitative relationships with functions but providing the information of the extent of involvement of side chain properties in the functions of interest.

For mutant G12W/H86V that gained the greatest activity increase of 4.98 ± 0.09 times (mean ± SD at n = 3) that of recombinant wild-type [14], the strand propensity decreased from 0.78 to 0.69 (H86V) with a slight increase in the helix propensity (corresponding to decrease in the index values). The same was true for mutant D15P/H86I with 2.65 ± 0.30 times activity increase. A similar result was observed in mutant G4L/D40I with 2.11 ± 0.29 times activity increase, due to strand decrease along with almost no change in helix (D40I). However, mutant V10S/R93G with 4.50 ± 0.07 times activity increase behaved differently with increased strand and simultaneous decrease in helix. It is worth noting that the single mutation of V10S alone increased the activity 2.96 ± 0.06 times, therefore, changes in the helix domain may have a more predominant effect on the inhibitory activity than mutations in the strand domain. The activity change due to mutation helix → strand in the strand domain in the sequence may be slight in this case.

It is well known that a single-point mutation of human lysozyme, namely I56T, has been identified as the origin of hereditary systemic amyloidosis [27]. The amyloidogenic nature of the lysozyme variants arises from a decrease in the stability of the native fold relative to partially folded intermediates. Accordingly, in a low population of soluble, partially folded species, the protein can aggregate in a slow and controlled manner to form amyloid fibrils. Similarly, sporadic amyloid angiopathy and intracerebral hemorrhage was reported in an elderly man due to cystatin C mutation [11]. In the case of human cystatin C, the decrease in strand along with an increase in helix might have prevented amyloidosis, despite the fact that helix change was not always as evident as in the case of lysozyme. Some inconsistency in the amyloidosis as a cause of inhibitory activity of human cystatin C in our mutation optimization [14] may be due to lack of the data of single-site mutation in the strand domain of the cystatin sequence. Unfortunately, the objective of that study [14] was for mutation optimization and not for investigation of the mechanism of amyloidosis. It has been reported that stefin B (HCB) readily formed amyloid [28], which may imply declined importance of the role being played by the binding site 2 in amyloidosis of HCC.

## Discussion

In a review on the quest to deduce protein function from sequences [29], the author stated that the searching of pattern databases would be more sensitive and selective than searching of sequence database. It was predicted that the sequence pattern databases, especially by comparing the pattern similarity, would play an increasingly important role, as the post-genome quest to assign functional information to raw sequence data gains pace [29]. Pattern similarity computation requires at least three residues in segments to represent a nonlinear curve, which is unlikely to be due to the effect of a single point mutation per se.

With regard to an apparent effect of the single residue mutations of hen lysozyme on substrate binding, the structural analysis by NMR of the position-62 mutant of hen lysozyme [18,30] found major changes in the chemical shift of back bone protons, especially in a loop region (positions 61–78), which contains W62 influencing the local folding. Similarly, Muraki et al. [20] reported that compared to the wild-type human lysozyme, the N-acetylglucosamine residue at subsite B of the L63 mutant markedly moved away from the 63^rd ^residue, with substantial loss of hydrogen-bonding interaction. In Figure 5 of Ref 17, involvement of not only Y63 but also W64 is evident. These results are supportive of the importance of pattern similarity of ≥ 3 residues, which are affected by single-residue mutation.

The predictability of the active and binding sites solely on the basis of protein sequences [31] may be useful for investigating the underlying mechanisms of unknown functions of human genes after translation to protein sequences. Usually, two or three essential residues are directly involved in the bond making and breaking steps leading to formation of enzyme catalysis; however, the removal of an essential group often does not abolish activity, but can significantly alter the catalytic mechanism [32]. T4 lysozyme was cited by Peracchi [32] as an example of the alteration of catalytic mechanisms; the lytic activity of lysozyme changed to that of a transglucosidase.

An approach utilizing the property of side chains in a sequence for identifying functional motifs has already been utilized in the computer-assisted selection of antigenic peptide sequences [33]. The authors stated that an antibody produced in response to a simple linear peptide with 7–9 residues in a protein would most likely recognize a linear epitope. Furthermore, this epitope must be solvent-exposed to be accessible to the antibody. In a large scale data mining study, Binkowski et al. [34] described the importance of local sequence and spatial surface patterns in inferring functional relationships of proteins. The general feature of protein structure that would correspond to these criteria could be turns or loop structures, which are generally found on the molecular surface connecting to other elements of secondary structure, and the area of high hydrophobicity, especially for those containing charged residues.

**Figure 8:**
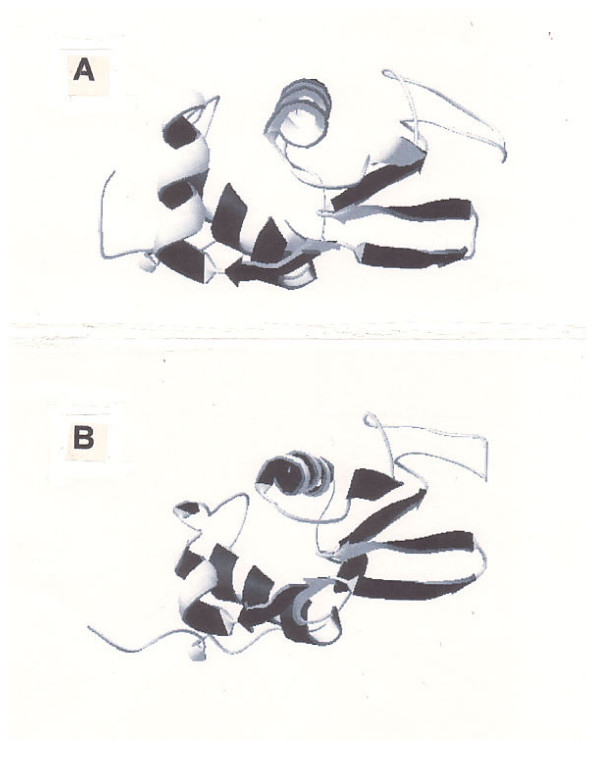
**An example of enzyme vs. non-enzyme**. Adopted from Figure 10.3b [36]. Fig. 8A: 1IWT human α-lactalbumin, Fig. 8B: 1B9O human lysozyme C using Swiss-PdbViewer (spdbv). Blast 2 sequences [1] showed that the identities and the positives between the two proteins were 35% and 55% respectively.

Successful identification of active sites of new-fold of CH-lysozyme using the HSS approach in this study suggests that this approach could be applied to query proteins translated from unknown RNA segments of the human genes against templates with known functions, when their 3D structure information is still unavailable. It has been shown that the inhibition of the papain family by cystatin is due to a tripartite wedge-shaped structure with a good supplement to the active site clefts of the enzyme [35]. Todd [36] stated that despite highly homologous relationship as seen in Figure 8, lysozyme functions as an O-glycosyl hydrolase, while α-lactalbumin lacks this activity and instead regulates the substrate specificity of galactosyltransferase. The active site of peptidoglycan lysis is disrupted in α-lactalbumin. The ESS computation showed that although a pattern equivalent to hen's D^53 ^exists in the form of T**E**YG/Y**D**YG, there is no residue equivalent to hen's E^35 ^around corresponding positions as in the form of HTSG/W**E**SG. Two side-chain carboxyl radicals are required for the lysozyme activity within the crevice between the helix and strand domains of the molecule belonging to C-type family [16]. According to Alvarez-Fernandez et al. [35], the three parts of the cystatin polypeptide chain included in the enzyme-binding domain are the N-terminal segment, a central loop-forming segment with motif QXVXG and second C-terminal loop typically containing a PW pair [31,37,38].

For multiple sequence alignment of an uncharacterized protein or peptide, many Web alignment servers are available for use [1], such as Blast and NPSA, as was done in this study. For classification of uncharacterized sequences, the PCS scatterplots are also useful as shown in Figures 1, 5 and 7. The PCA demonstrated the classifying capacity superior to that of distance-based cluster analysis [39]. The PCS is more flexible than cluster analysis as different pattern similarity patterns can be drawn by rotating the reference segment for searching. It implies that similarity is not always [1 – dissimilarity]. This difference resulted in the possibility of selecting outliers, which is critical in deriving true classes or ranking [40]. Most of the currently available peptide QSAR, such as the method of Hellberg et al. [4], intends to be based on whole sequence data. The new HSS approach reported in this study could be just the beginning of more detailed, reliable peptide QSAR to be developed in the future. Analysis of a variety of bioactive proteins contributing to human health is a potential future application of the HSS software package as well as multifunctional PCS. Considering the multifunctional nature of human diseases, the functionality of food proteins also can be manipulated based on combinations of bioactive segments in different or even single natural protein sequences. Therefore, for an uncharacterized protein or peptide, a new plan is proposed: (1) A reference sequence is chosen from multiple sequence alignment (MSA) as discussed above; PCS scattergrams would assist this selection in addition to BLAST search. (2) Based on segments with high similarity in MSA, segments to be used for search are selected within the reference sequence. Then, (3) HSS search is conducted to identify functional segments in the uncharacterized sequence. (4) From the above PCS computation, important PC scores are screened (PCA is a subroutine subprogram of PCS). (5) Regression neural networks are conducted using selected PC scores as input variables as exemplified in our lactoferricin derivative study [6]. (6) RCG would be useful for confirming the HSS data and also to find the best segment or sequence as exemplified in our HCC mutation [14].

One of the original purposes of our new approach in unsupervised data mining was to verify the hypothesis that there might be adaptability of different human cystatins to better inhibit different human cathepsins [41]. This hypothesis has not been fully pursued in the past, probably because of costly separation of pure cystatins and cathepsins. An advantage of our approach is to derive potential hypothesis for enzyme/substrate interactions exclusively from their sequence data. Although the verification of those hypotheses may need to await future 3D-structure study, it is important that most of the useful QSAR data could become available, which would promote the functional mechanism study based on 3D structure. However, we admit that more examples of application should be performed in the future to more thoroughly verify and establish this method for predicting functions based on sequences. This work is underway in our laboratory.

## Conclusion

Although the importance of pattern similarities of motifs with 20–30 residues as a whole has been reported for peptide QSAR in the past, the importance of a search for segments with three or more residues as functional sites of protein sequences has not been investigated. Lysozymes and cystatins were used as examples of proteins to demonstrate the capacity of segment pattern similarity analysis to predict functions, such as active and binding sites, amyloidosis and thermostability as a tool for quantitative functional sequence analysis.

## Methods

### Amino acid sequences of proteins

Multiple sequence alignments of lysozymes were conducted using the Network Protein Sequence Analysis of Pôle Bio-Informatique Lyonnais [42] based on Clustal W. Similarly, multiple sequence alignments were obtained for human cystatins A (HCA), B (HCB) and C (HCC) and hen egg white cystatin (EWC) as well as for papain as host proteases of cysteine protease inhibitors, i.e. cystatins. For PCS analysis, a total of 17 cystatins were used: human A, B, C, D, E, F, M, S, SA, SN, hen (EWC), bovine, ratC, mouseC, Chum salmon, Rainbow trout and carp.

### Principal components similarity analysis of protein sequences

The method described in the previous papers [9,39] was followed. Principal components analysis (PCA) was modified to principal components similarity (PCS) by incorporating linear regression of PC scores to be able to account for more than three PC scores on a 2D scatter plot. The PCS was then modified to apply to peptide sequences.

### Homology similarity search

Homology similarity search (HSS) was conducted as reported previously [9]. The similarity constant used in this study is eventually a correlation coefficient [43]. A preliminary study was carried out by changing the size of segment (normally 3–7) flanking the potential functional position to determine the most appropriate size of segment in differentiating the functional site from other segments within the sequence of lysozymes and cystains. The property indices used for amino acid side chains were hydrophobicity, charge, propensities of α-helix, β-strand and β-turn, hydrogen bonding, and bulkiness as reported previously [14,44]. Segments with pattern similarity close to 1.0 and average values similar to that of the reference segment were sought within each gapped sequence.

All software used in this study along with the instructions on how to use the computer programs are available in the form of ftp files on the Web [45] to download to PC computers.

## List of abbreviations

EWC Egg white cystatin or hen cystatin.

HCC Human cystatin C.

HCA Human cystatin A or stefin A.

HCB Human cystatin B or stefin B.

HSA Homology similarity analysis: the PCS software was modified to compute pattern similarity constants and average side-chain property index values of segments in sequences [6].

HSS Homology similarity search: A step-wise search program initiated from N-terminus of query sequences by shifting the search unit (reference segment) towards C-terminus based on similar segments in terms of pattern similarity constant and average property values compared to those of template sequences [9].

MIC Minimum inhibitory concentration.

PCA Principal components analysis.

PCS Principal components similarity: PCA modified for multi-functional variables using linear regression of deviation of PC scores on the reference PC scores. Scatter plot is drawn as slope vs. coefficient of determination (r^2^) [44].

RCG Random-centroid optimization of site directed mutagenesis.

QSAR Quantitative structure-activity relationships.

## Authors' contributions

EL participated in laboratory investigation to verify the hypothesis set in this study, while JD was taking care of the computer programming. SN was responsible mainly for the creation and application of software used in this study. All authors read and approved the final manuscript.
